# Effect of an Exercise Intervention Program on BMI and Distance Covered in the 6 Min Walking Test (6MWT) in Obese Children

**DOI:** 10.3390/children11121473

**Published:** 2024-11-30

**Authors:** Ourania Papadopoulou, Evangelia Desli, Elisavet-Anna Chrysochoou, Christos Kogias, Vasileios Liakos, Athina Sopiadou, Petrina Vantsi, Ilias Kallistratos, Paris Iakovidis, Kiriaki Tsiroukidou, Elpis Hatziagorou

**Affiliations:** 1 Department of Physiotherapy, International Hellenic University, 57400 Thessaloniki, Greece; 2 Pediatric Pulmonology and CF Unit, 3rd Department of Pediatrics, Hippokration Hospital, Aristotle University of Thessaloniki, 54124 Thessaloniki, Greecechrkog4@gmail.com (C.K.); liakospediatrics@gmail.com (V.L.); ktsiroukidou@gmail.com (K.T.)

**Keywords:** physical activity, 6-minute walking test, BMI, obesity, children, exercise, physiotherapy

## Abstract

Background/Objectives: Childhood obesity appears to be an alarmingly growing global threat. Current evidence has shown that obesity can be successfully managed with interventions targeting movement skills, motor coordination and physical activity. However, data concerning physiotherapy practice are limited. We aimed to assess the impact of therapeutic exercise on the 6-minute walk test (6-MWT) in obese children and adolescents. Methods: A total of 31 obese children and adolescents (BMI > 30 kg/m^2^), 18 males and 13 females, aged from 8 to 18 years, were enrolled. Two study groups were formed: the control group (Group A), comprising ten children; and the intervention group (Group B), comprising 21 children. Daily exercise habits were obtained via interview; anthropometric data (weight, height and BMI) were recorded; and 6-MWT parameters, blood pressure and oxygen saturation (SaO_2_) were assessed before and after a 4-month intervention program consisting of breathing and aerobic exercises. Results: There was a statistically significant increase in 6-minute walk distance (+43.34, *p* < 0.001) and an improvement in body weight (*p* < 0.01), blood pressure (*p* < 0.01) and oxygen saturation (*p* < 0.03) in Group B after the 4-month training program. Conclusions: All results highlight the potential of physical exercise in clinical practice to improve functional status and achieve weight loss. Future randomized controlled trials, including individualized therapeutic exercise programs in larger samples of obese children, are much anticipated.

## 1. Introduction

Childhood obesity is an alarmingly growing threat globally, with a fourfold increase in prevalence over the past forty years [1,2]. At present, over 340 million children worldwide are overweight or obese [3]. Obesity appears to be a multifactorial disease related to genetic causes and social parameters. Physical inactivity and a sedentary lifestyle are considered significant risk factors and show a growing trend in the pediatric population. According to numerous studies, the pandemic resulted in a devastating increase in screen time among children and adults, 67% and 51%, respectively. Children aged from 6 to 10 years have demonstrated the most significant increase in screen time, with 1.4 h a day, followed by adolescents with 0.9 h and young children with 0.6 h a day [4]. It is estimated that each additional hour per day spent in front of a television screen seems to raise obesity prevalence by 2%, taking into consideration lousy eating habits and lack of exercise [5]. In addition, during the last three decades, no country has won the battle against significantly decreasing obesity rates [6]. Despite numerous efforts to define obesity cut-off weight points in children throughout the years, and due to the poor understanding of its pathophysiology, there is no doubt that obese children suffer from both short- and long-term comorbidities. It seems that no organ system is left unaffected while posing a greater risk of human morbidity and cardiovascular-related mortality [7]. There is a strong correlation between childhood obesity and several medical conditions, including arterial hypertension, diabetes mellitus type 2, fatty liver disease, precocious puberty, polycystic ovarian syndrome, menstrual disorders, benign intracranial hypertension, musculoskeletal and psychological problems. Focusing on respiratory consequences, obese children tend to show a higher frequency of obstructive sleep apnea syndrome [8], asthma [9], alveolar hypoventilation [10] and significant restriction of respiratory capacity [11] in comparison with their normal-weight peers.

It is common practice for medical studies on childhood and adolescent obesity to include lung volume measurements, such as spirometry, in terms of pulmonary function tests (PFTs), but not during exercise. A walking test is a submaximal exercise activity that may provide a safe and practical alternative to evaluate functional status [12], monitor any therapeutic effect [13] and assess prognosis [14]. The 6 min walk test (6MWT) procedure involves measuring the distance one can walk in a 6-minute timeframe, rendering it one of the most used exercise tests in clinical practice. Among its numerous advantages, we must highlight safety, simplicity, inexpensiveness, good tolerance and standardization, consistent validity and reproducibility in obese subjects [14]. It has also been reported that the 6MWT correlates with patients’ functional level in daily activities [15] and can be a good predictor of peak oxygen uptake (VO_2peak_) in diseased patients [16,17]. In 2002, the American Thoracic Society (ATS) published detailed guidelines for the test that delineate its indications and provide instructions for achievement and parameters for interpretation [18]. Nevertheless, most of the studies that used the 6MWT were conducted in adults, posing difficulty in developing reference equations for the pediatric population.

Energy balance is the core target of any intervention regarding childhood obesity and is defined as the difference between energy intake and energy expenditure. As a result, excess adiposity seems to be the direct consequence of energy intake surpassing energy expenditure. It is common sense that obesity management can be achieved with two approaches, either using nutritional education to decrease energy intake or physical activity to maximize energy expenditure [19]. Physical activity is the cornerstone of preventing and treating childhood obesity. Skeletal muscle is a powerful oxidizer of fat, and its role in maintaining a healthy daily fat balance is vital. Moreover, it seems there are several confounding factors of childhood obesity, including gender, age, genetics, psychological and environmental parameters (e.g., school policies), lifestyle and parents’ work-related demands [20]. Consequently, gathering a multidisciplinary team and adopting a multicomponent intervention are paramount to maximizing any beneficial effect [21,22,23]. The current evidence on childhood obesity management focuses on enhancing the fundamentals of movement skills, motor coordination and physical activity. It is well established that children’s level of motor skills is strongly associated with a higher chance of engaging in advanced physical activity levels [24]. Obese patients tend to exhibit lower levels of balance, agility, speed, coordination and motor skills, magnifying their inability to follow any physical activity recommendations and benefit from any exercise [25,26,27]. Considering all the data mentioned above, physiotherapists have an apparent crucial role in motor-skill and physical-activity quality among obese subjects [28].

Nevertheless, there is a knowledge gap in terms of appropriate exercise protocols for childhood obesity’s optimal management. All studies published have continuously highlighted the need for evidence-based guidelines to address effective exercise interventions for childhood obesity [29]. There are no studies on the effect of an exercise intervention program on 6MWT performance in obese children. The present study aims to assess the impact of a therapeutic exercise program on 6MWT performance in obese children and adolescents (BMI > 30 kg/m^2^).

## 2. Method

### 2.1. Study Population

Thirty-one children and adolescents aged from 8 to 18 years with obesity and who were followed in the Paediatric Endocrinology and Paediatric Pulmonology Unit of a tertiary University Hospital participated in the study. The children were randomized into the control group (Group A, 10 patients) and the intervention group (Group B, 21 patients). A 4-month exercise program, which included breathing and aerobic exercises, was applied to the intervention group (Group B), while the control group (Group A) did not change its daily physical activity practice over the 4 months. All the children (Group A and B) showed sedentary behavior at baseline.

To determine obesity, participants’ Body Mass Index (BMI) in regard to age and sex-specific BMI curves were used, with obese children being those with BMI > 30 kg/m^2^ [30].

Before commencing the 4-month exercise intervention program, all participants and parents or legal guardians were fully informed, providing written consent. At the beginning and the end of the program, the patients underwent a clinical examination, anthropometric measurements, spirometry and the 6MWT.

Exclusion criteria included acute or chronic diseases, respiratory diseases, or any neurological or physical limitations that may have interfered with their ability to perform spirometry or the 6MWT.

The study was conducted following the Declaration of Helsinki and approved by the Ethics Committee of International Hellenic University (04/20.04.2022).

### 2.2. Anthropometric Evaluation

During the first and last intervention visit, the participants’ height (centimeters, cm) and weight (kilograms, kg) were measured using standardized procedures and identical equipment. Standing height was measured with a Raven Minimeter (Raven Equipment Limited, Essex, UK) to the nearest 0.1 cm, after children had removed their shoes, and body weight was measured to the nearest 0.1 kg with a Seca weighing scale (Seca, Hanover, MD, USA). All height measurements were taken by a trained nurse using the same stadiometer. The Body Mass Index (BMI) was then calculated (kg/m^2^).

### 2.3. Six-Minute Walk Test (6MWT)

The six-minute walk test (6MWT) is a standardized assessment that evaluates patients’ functional exercise capacity. It is performed in a confined, 30 m long corridor, following the American Thoracic Society guidelines 2002. The course should be marked at 3 m intervals to provide a reference point for the patient. A cone should be placed at each turnaround point for the patient to follow. A starting line should be drawn on the floor with brightly colored tape to demarcate the beginning and end of each 60 m lap. Patients are instructed to walk as quickly as possible for 6 min, while only standardized phrases of encouragement are used during the test. Identical procedures are used before and after training to maintain consistency.

Heart rate (HR), systolic blood pressure (SBP), diastolic blood pressure (DBP) and oxygen saturation are measured twice: once before the 6MWT (after 15 min of rest) and once after the completion of the test. A digital sphygmomanometer (Omron M2 Intelli IT) and an oximeter (Nonin Wristox2 3150 (Plymouth, MA, USA)) are used to take these measurements. In addition, the total distance walked during the test is recorded for each patient.

Perceived fatigue and shortness of breath are monitored at the beginning and end of the 6MWT using the Borg scale of perceived exertion (category range, 0–10). This scale is used to assess the patient’s level of perceived exertion during the test. Monitoring these symptoms helps evaluate the patient’s physical condition [18].

### 2.4. Exercise Training Program

The intervention group was subjected to a 4-month exercise program, which included breathing and aerobic exercises [18,31]. The program consisted of specific breathing exercises, such as diaphragmatic breathing and breathing coordination exercises, which improved respiratory muscle strength and endurance. Different aerobic exercises were included to increase the heart rate to the extent that it makes it somewhat breathless and sweaty, like step-up exercises, walking, aerobic steps, walking stairs up, on-spot jogging and jumping jack exercises. Moreover, different body positions (prone, supine, quadrupedal, sitting and standing) were used during the aerobic exercise. Each session lasted 45 min.

The children were encouraged to perform physical therapy twice daily (morning and evening), from three to five days a week. The instructor demonstrated the first session to the participants, aiming to give them a better understanding of the exercise program. A video, including a complete exercise program from all positions, was administered to the family to ensure the correct implementation of the exercises. Compliance with the therapeutic program was monitored via telephone contact and online sessions during the study. Follow-up home visits were also conducted twice monthly. The duration of the intervention was four months.

### 2.5. Statistical Analysis

Statistical analysis was performed by SPSS, version 25.0 (SPSS, Chicago, IL, USA). Continuous variables were presented as mean ± standard deviation (SD). Data were checked for normality before analysis. A paired *t*-test was used to evaluate the significance of mean differences in variables recorded before and after exercise intervention. For all statistical tests, *p* ≤ 0.05 was considered statistically significant.

## 3. Results

A total sample of 31 obese children and adolescents participated in the study. The participants were 18 males and 13 females, with a mean age of 11.94 years. The mean weight was 88.81 kg, the mean height was 152.58 cm and the mean BMI was 38.37 kg/m^2^. The children were divided into two groups: the control group (A; ten children) and the intervention group (B; 21 children). The baseline characteristics of the two groups were comparable, as shown in Table 1. The results of the exercise intervention on the 6MWT and the Borg scale’s main parameters in both groups are shown in Table 2 and Table 3.

### 3.1. Primary Outcomes

There was a statistically significant increase in 6-minute walk distance (+43.34 m, *p* < 0.001) in the intervention group, as measured by the total distance walked and steps, after the 4-month training program with physical exercise (Table 2).

### 3.2. Secondary Outcomes

The intervention group’s body weight decreased by 2.21 kg (*p* < 0.01). Blood pressure and oxygen saturation parameters were also positively affected in group B. There was a statistically significant reduction in systolic (−8.24 mmHg, *p* < 0.01) blood pressure and an increase in oxygen saturation % (+1.28, *p* < 0.01). Finally, the perceived exertion, as measured by the Borg scale, improved (+0.52, *p* <0.001) after the exercise program (Table 2).

## 4. Discussion

The 4-month training program with physical and breathing exercises significantly increased the cardiopulmonary capacity of obese children, as measured by the 6MWT. A statistically significant improvement in BMI, weight, blood pressure and oxygen saturation was also recorded.

The findings of our 4-month training program suggest that physical and breathing exercises can improve cardiopulmonary function and body weight.

Obesity affects nearly every organ system, resulting in an increased risk of morbidity and cardiovascular system-related mortality. Overweight and obese children are at high risk of hypercholesterolemia, dyslipidemia and hypertension, while hyperinsulinism, insulin resistance, type 2 diabetes mellitus and menstrual irregularity are among the most common endocrine system disorders. In addition, children with obesity are more likely to present musculoskeletal problems. Moreover, according to recent data, they are more susceptible to mental health issues such as depression, low self-esteem, distorted body image and eating disorders [32].

Regarding the respiratory system, obesity significantly affects the pulmonary mechanics, reducing lung volumes and increasing airway resistance. Thoracic compliance is attenuated in obese patients due to the physical limitation on chest wall expansion and diaphragm contraction caused by excessive abdominal adipose tissue. Lung compliance is also reduced because of the increase in pulmonary blood flow and the closure of the peripheral airways. Expiratory reserve volume (ERV) is the first parameter affected in obese patients, resulting in a reduction in functional residual capacity (FRC), while vital capacity (VC), forced vital capacity (FVC) and forced expiratory volume in 1 s (FEV1) are slightly affected [33]. Obese children tend to show reduced functional capacity even in low-intensity exercises in comparison with their normal-weight counterparts. They exhibit poorer performance in low-intensity exercise tests and show elevated hemodynamic parameters (BP and HR) before and after the exercise [34]. Obese children are also believed to be future asthmatic individuals. The airway inflammation and narrowing probably explain this. Obesity tends to correlate with higher hospitalization rates for asthma exacerbation and lower response rates to inhaled corticosteroids [18]. Obstructive sleep apnea syndrome (OSA) is another common condition in this group; 60–70% of OSA patients are obese, and the risk of OSA rises with the increase in BMI. Apart from physical upper airway obstruction, obese people show reduced airway caliber and thus have a high risk of airway collapse. Pulmonary vascular disease, respiratory tract infections and acute respiratory distress syndrome are other conditions strongly related to obesity, with a higher prevalence among severely obese subjects [33].

Given those mentioned above, functional capacity must be examined in obese children to identify the potential risks for respiratory and cardiovascular disease. The six-minute walk test (6MWT) is a submaximal exercise testing method that examines cardiovascular capacity and can be well-tolerated by most individuals, even those with moderately severe impairment [18]. One of the main advantages of the 6MWT is its simplicity of use, requiring a 100 ft hallway but no exercise equipment or advanced training for technicians. It evaluates the global and integrated responses of all the systems involved during exercise, including the pulmonary and cardiovascular system, systemic circulation, peripheral circulation, blood, neuromuscular units and muscle metabolism. However, there are some limitations regarding its use. The six-minute walk test does not diagnose the cause of dyspnea upon exertion or evaluate the causes or mechanisms of exercise limitation, so the information provided should be considered complementary to the cardiopulmonary exercise testing (CPET), which is the gold-standard method to evaluate cardiopulmonary capacity [18]. Another limitation of the method is that the outcome of the 6MWT can be affected by a variety of parameters, such as age, sex, height, weight, BMI, waist circumference (WC), hip circumference (HC), estimated fat-free mass (FFM) and ethnic background [35,36]. Consequently, there should be many statistical adjustments to evaluate the results and reach a conclusion.

Focusing on obese subjects, the 6MWT offers the opportunity to evaluate the functional status in a timely, practical and economically advantageous manner. It can be used to evaluate the outcome of an intervention program, monitor treatment effectiveness and establish prognosis. Morinder et al. demonstrated that the 6MWT has good reproducibility and known group validity and can be recommended in clinical practice in obese children and adolescents [14]. Another study by Makni et al. suggested that the 6MWT can predict maximal fat oxidation rate (FATmax) when formal tests of exercise capacity and gas exchange analysis are unavailable or impractical. The subject could be assessed over time, which is essential to understanding the progression of a disease or the changes resulting from medical interventions [12,13]. In a larger study with 309 participants, the 6MWT was used to assess the functional capacity of obese/overweight children compared to their normal-weight peers. Most participants (95%) expressed willingness to complete the test; unsurprisingly, the 6MWT distance was lower in the overweight/obese group.

The findings of our 4-month training program suggest that physical and breathing exercises can improve cardiopulmonary function and improve body weight. Diaphragmatic breathing and breathing coordination exercises improve inspiratory muscle strength and exercise tolerance. The results of our study are compatible with findings from previous trials. In the study by P. Delgado-Floody et al. [37], significant improvements in the cardiorespiratory capacity, anthropometric variables and blood pressure levels of overweight and obese children were observed after a 28-week high-intensity interval training program. The distance covered in the 6 min walk test improved significantly for the overweight and obese girls (*p* < 0.05) and obese boys, while there were also significant reductions in body mass index (*p* < 0.001). Kaeotawee et al. [38] came to the same conclusions, as they revealed that functional fitness, as measured by the 6-MWT, in children and adolescents with obesity was positively affected (*p* < 0.002) after a threshold inspiratory muscle training program for 8 weeks. Finally, a recent systematic review and meta-analysis showed that physical activity could effectively improve the body weight, maximal oxygen consumption and hemodynamic parameters of obese children and adolescents, with moderate-to high-intensity physical activity demonstrating the most significant impact on intervention outcomes [39].

### Limitations of the Study

One of the limitations of our study is the lack of obesity classification. The severity of obesity strongly affects overall performance and modifies the type of intervention. Moreover, the parameters used in our study included weight, height and BMI. It is essential to assess body composition more accurately by using waist circumference (WC), hip circumference (HC), waist–hip ratio (WHR) and estimated fat-free mass (FFM) to obtain precise data concerning cardiopulmonary capacity in obese children. Another limitation of our study is the small size of the participants; despite statistical adjustments, it contributes to a small sampling bias.

## 5. Conclusions

The 4-month training program with physical exercise significantly increased the cardiopulmonary capacity of obese children, as measured by the 6MWT. A statistically significant improvement in BMI, weight, blood pressure and oxygen saturation was also recorded. These results suggest that physical and breathing exercises, supplementary to dietary interventions, could be used in clinical practice to improve functional status and achieve weight loss. Future randomized controlled trials assessing individualized and structured therapeutic exercise programs in a larger sample of obese children are much anticipated.

## Figures and Tables

**Table 1:** Comparison of baseline parameters between the control and intervention groups.

	Control Group		Intervention Group		
	N	Mean (SD)	N	Mean (SD)	*p*
Weight, kg	10	94.24 (32.18)	21	83.24 (21.44)	0.141
BMI, kg/m^2^	10	38.58 (8.11)	21	34.37 (5.47)	0.085
Systolic blood pressure, mmHg	10	128.1 (13.74)	21	138.19 (10.02)	0.027
Diastolic blood pressure, mmHg	10	80.6 (6.94)	21	81.43 (12.32)	0.845
Pulse rate, pulse/min	10	113.2 (19.34)	21	119.57 (22.54)	0.449
Oxygen saturation, %	10	97.8 (0.421)	21	96.86 (1.65)	0.022
Total distance walked, m	10	374.1 (60.09)	21	386.95 (53.9)	0.554
STEPS	10	561 (120.99)	21	680.43 (66.79)	0.013
Borg scale	10	0.9 (0.76)	21	0.76 (0.83)	0.132
Level of Effort Scale	10	2.2 (0.58)	21	0.95 (1.02)	0.214
Severity of dyspnea	10	1 (0.67)	21	0.95 (0.38)	0.095

**Table 2:** Comparison of anthropometric, 6MWT and BORG scale parameters for the intervention group. Significant changes were noted in weight, BMI, systolic blood pressure, oxygen saturation, total distance walked and BORG scale parameters.

	Intervention Group					
		Baseline Measurement		2nd Measurement		
	N	Mean	SD	Mean	SD	*p*-Value
Weight, kg	21	83.24	21.44	80.97	21.82	0.002
BMI, kg/m^2^	21	34.37	5.47	32.70	6.07	<0.001
Systolic blood pressure, mmHg	21	138.19	10.02	129.95	9.83	<0.001
Diastolic blood pressure, mmHg	21	81.43	12.32	78.14	10.05	0.246
Pulse rate, pulse/min	21	119.57	22.54	113.19	20.25	0.130
Oxygen saturation, %	21	96.86	1.65	98.14	0.79	<0.001
Total distance walked, m	21	386.95	53.9	430.29	55.83	<0.001
STEPS	21	680.43	66.79	746.81	32	<0.001
Borg scale	21	0.76	0.83	0.24	0.63	0.005
Level of Effort Scale	21	0.95	1.02	0.29	0.64	0.002
Severity of dyspnea	21	0.95	0.38	0.33	0.48	0.001

**Table 3:** Change in anthropometric, 6MWT and BORG scale parameters for the control group.

	Control Group					
		Baseline Measurement		2nd Measurement		
	N	Mean	SD	Mean	SD	*p*-Value
Weight, kg	10	94.24	32.18	98.32	43.05	0.140
BMI, kg/m^2^	10	38.58	8.11	41.00	10.43	0.141
Systolic blood pressure, mmHg	10	128.1	13.75	127.9	15.42	0.959
Diastolic blood pressure, mmHg	10	80.6	6.95	82.5	7.44	0.384
Pulse rate, pulse/min	10	113.2	19.34	115.3	16.73	0.385
Oxygen saturation, %	10	97.8	0.42	97.9	0.57	0.317
Total distance walked, m	10	374.1	601	387.8	46.24	0.151
STEPS	10	561	121	609	125.45	0.114
Borg scale	10	0.9	1,2	0.8	0.92	0.317
Level of Effort Scale	10	2.2	2.97	2.1	2.92	0.317
Severity of dyspnea	10	1	0.67	0.9	0.57	0.317

## Data Availability

The raw data supporting the conclusions of this article will be made available by the authors upon request.

## References

[1] Bentham J., Di Cesare M., Bilano V., Bixby H., Zhou B., Stevens G.A., Riley L.M., Taddei C., Hajifathalian K., Lu Y. (2017). Worldwide Trends in Body-Mass Index, Underweight, Overweight, and Obesity from 1975 to 2016: A Pooled Analysis of 2416 Population-Based Measurement Studies in 128·9 Million Children, Adolescents, and Adults. Lancet.

[2] Skinner A.C., Ravanbakht S.N., Skelton J.A., Perrin E.M., Armstrong S.C. (2018). Prevalence of Obesity and Severe Obesity in US Children, 1999–2016. Pediatrics.

[3] World Health Organization (2020). Basic Documents: Forty-Ninth Edition (Including Amendments Adopted Up to 31 May 2019).

[4] Choi E.J., King G.K.C., Duerden E.G. (2023). Screen Time in Children and Youth during the Pandemic: A Systematic Review and Meta-Analysis. Glob. Pediatr..

[5] Anderson P.M., Butcher K.F. (2006). Childhood Obesity: Trends and Potential Causes. Futur. Child..

[6] Ng M., Fleming T., Robinson M., Thomson B., Graetz N., Margono C., Mullany E.C., Biryukov S., Abbafati C., Abera S.F. (2014). Global, Regional, and National Prevalence of Overweight and Obesity in Children and Adults during 1980-2013: A Systematic Analysis for the Global Burden of Disease Study 2013. Lancet.

[7] Ebbeling C.B., Pawlak D.B., Ludwig D.S. (2002). Childhood Obesity: Public-Health Crisis, Common Sense Cure. Lancet.

[8] Narang I., Mathew J.L. (2012). Childhood Obesity and Obstructive Sleep Apnea. J. Nutr. Metab..

[9] Papoutsakis C., Priftis K.N., Drakouli M., Prifti S., Konstantaki E., Antonogeorgos G., Chondronikola M., Matziou V. (2013). Childhood Overweight/Obesity and Asthma: Is There a Link? A Systematic Review of Recent Epidemiologic Evidence. J. Acad. Nutr. Diet..

[10] Verhulst S.L., Schrauwen N., Haentjens D., Rooman R.P., Van Gaal L., De Backer W.A., Desager K.N. (2007). Sleep-Disordered Breathing and the Metabolic Syndrome in Overweight and Obese Children and Adolescents. J. Pediatr..

[11] El-Baz F.M., Abdelaziz E.A., Abdelaziz A.A., Kamel T.B., Fahmy A. (2009). Impact of Obesity and Body Fat Distribution on Pulmonary Function of Egyptian Children. Egypt. J. Bronchol..

[12] Makni E., Moalla W., Trabelsi Y., Lac G., Brun J.F., Tabka Z., Elloumi M. (2012). Six-Minute Walking Test Predicts Maximal Fat Oxidation in Obese Children. Int. J. Obes..

[13] Elloumi M., Makni E., Ounis O.B., Moalla W., Zbidi A., Zaoueli M., Lac G., Tabka Z. (2011). Six-Minute Walking Test and the Assessment of Cardiorespiratory Responses during Weight-Loss Programmes in Obese Children. Physiother. Res. Int..

[14] Morinder G., Mattsson E., Sollander C., Marcus C., Larsson U.E. (2009). Six-Minute Walk Test in Obese Children and Adolescents: Reproducibility and Validity. Physiother. Res. Int..

[15] Geiger R., Strasak A., Treml B., Gasser K., Kleinsasser A., Fischer V., Geiger H., Loeckinger A., Stein J.I. (2007). Six-Minute Walk Test in Children and Adolescents. J. Pediatr..

[16] Cahalin L.P., Mathier M.A., Semigran M.J., Dec G.W., DiSalvo T.G. (1996). The Six-Minute Walk Test Predicts Peak Oxygen Uptake and Survival in Patients with Advanced Heart Failure. Chest.

[17] Carter R., Holiday D.B., Stocks J., Grothues C., Tiep B. (2003). Predicting Oxygen Uptake for Men and Women with Moderate to Severe Chronic Obstructive Pulmonary Disease. Arch. Phys. Med. Rehabil..

[18] Crapo R.O., Casaburi R., Coates A.L., Enright P.L., MacIntyre N.R., McKay R.T., Johnson D., Wanger J.S., Zeballos R.J., Bittner V. (2002). ATS Statement: Guidelines for the Six-Minute Walk Test. Am. J. Respir. Crit. Care Med..

[19] Nga V.T., Dung V.N.T., Chu D.T., Tien N.L.B., Van Thanh V., Ngoc V.T.N., Hoan L.N., Phuong N.T., Pham V.H., Tao Y. (2019). School Education and Childhood Obesity: A Systemic Review. Diabetes Metab. Syndr..

[20] Sahoo K., Sahoo B., Choudhury A.K., Sofi N.Y., Kumar R., Bhadoria A.S. (2015). Childhood Obesity: Causes and Consequences. J. Fam. Med. Prim. Care.

[21] Al-Khudairy L., Loveman E., Colquitt J.L., Mead E., Johnson R.E., Fraser H., Olajide J., Murphy M., Velho R.M., O’Malley C. (2017). Diet, Physical Activity and Behavioural Interventions for the Treatment of Overweight or Obese Adolescents Aged 12 to 17 Years. Cochrane Database Syst. Rev..

[22] Colquitt J.L., Loveman E., O’Malley C., Azevedo L.B., Mead E., Al-Khudairy L., Ells L.J., Metzendorf M.I., Rees K. (2016). Diet, Physical Activity, and Behavioural Interventions for the Treatment of Overweight or Obesity in Preschool Children up to the Age of 6 Years. Cochrane Database Syst. Rev..

[23] Mead E., Brown T., Rees K., Azevedo L.B., Whittaker V., Jones D., Olajide J., Mainardi G.M., Corpeleijn E., O’Malley C. (2017). Diet, Physical Activity and Behavioural Interventions for the Treatment of Overweight or Obese Children from the Age of 6 to 11 Years. Cochrane Database Syst. Rev..

[24] Liang J., Matheson B.E., Kaye W.H., Boutelle K.N. (2014). Neurocognitive Correlates of Obesity and Obesity-Related Behaviors in Children and Adolescents. Int. J. Obes..

[25] Lubans D.R., Morgan P.J., Cliff D.P., Barnett L.M., Okely A.D. (2010). Fundamental Movement Skills in Children and Adolescents: Review of Associated Health Benefits. Sport. Med..

[26] Stodden D.F., Langendorfer S.J., Goodway J.D., Roberton M.A., Rudisill M.E., Garcia C., Garcia L.E. (2008). A Developmental Perspective on the Role of Motor Skill Competence in Physical Activity: An Emergent Relationship. Quest.

[27] Tsiros M.D., Coates A.M., Howe P.R.C., Grimshaw P.N., Buckley J.D. (2011). Obesity: The New Childhood Disability?. Obes. Rev..

[28] Long J. (2019). European Region of the WCPT Statement on Physiotherapy in Primary Care. Prim. Health Care Res. Dev..

[29] Milne N., Choy N.L., Leong G.M., Hughes R., Hing W. (2016). Child Obesity Service Provision: A Cross-Sectional Survey of Physiotherapy Practice Trends and Professional Needs. Aust. J. Prim. Health.

[30] Hampl S.E., Hassink S.G., Skinner A.C., Armstrong S.C., Barlow S.E., Bolling C.F., Edwards K.C.A., Eneli I., Hamre R., Joseph M.M. (2023). Clinical Practice Guideline for the Evaluation and Treatment of Children and Adolescents with Obesity. Pediatrics.

[31] Truong K., Park S., Tsiros M.D., Milne N. (2021). Physiotherapy and Related Management for Childhood Obesity: A Systematic Scoping Review. PLoS ONE.

[32] Moradi M., Mozaffari H., Askari M., Azadbakht L. (2022). Association between Overweight/Obesity with Depression, Anxiety, Low Self-Esteem, and Body Dissatisfaction in Children and Adolescents: A Systematic Review and Meta-Analysis of Observational Studies. Crit. Rev. Food Sci. Nutr..

[33] Shah N.M., Kaltsakas G. (2023). Respiratory Complications of Obesity: From Early Changes to Respiratory Failure. Breathe.

[34] Giontella A., Tagetti A., Bonafini S., Marcon D., Cattazzo F., Bresadola I., Antoniazzi F., Gaudino R., Cavarzere P., Montagnana M. (2024). Comparison of Performance in the Six-Minute Walk Test (6MWT) between Overweight/Obese and Normal-Weight Children and Association with Haemodynamic Parameters: A Cross-Sectional Study in Four Primary Schools. Nutrients.

[35] Makni E., Elloumi A., Ben Brahim M., Moalla W., Tabka Z., Chamari K., Elloumi M. (2020). Six-Minute Walk Distance Equation in Children and Adolescents with Obesity. Acta Paediatr..

[36] Makni E., Hachana Y., Elloumi M. (2023). Allometric Association between Six-Minute Walk Distance and Both Body Size and Shape in Young Obese Girls. Children.

[37] Delgado-Floody P., Espinoza-Silva M., García-Pinillos F., Latorre-Román P. (2018). Effects of 28 Weeks of High-Intensity Interval Training during Physical Education Classes on Cardiometabolic Risk Factors in Chilean Schoolchildren: A Pilot Trial. Eur. J. Pediatr..

[38] Kaeotawee P., Udomittipong K., Nimmannit A., Tovichien P., Palamit A., Charoensitisup P., Mahoran K. (2022). Effect of Threshold Inspiratory Muscle Training on Functional Fitness and Respiratory Muscle Strength Compared to Incentive Spirometry in Children and Adolescents with Obesity: A Randomized Controlled Trial. Front. Pediatr..

[39] Wang C., Tian Z., Hu Y., Luo Q. (2023). Physical Activity Interventions for Cardiopulmonary Fitness in Obese Children and Adolescents: A Systematic Review and Meta-Analysis. BMC Pediatr..

